# Flavonol Technology: From the Compounds’ Chemistry to Clinical Research

**DOI:** 10.3390/molecules30153113

**Published:** 2025-07-25

**Authors:** Tomasz Przybylski, Joanna Czerniel, Jakub Dobrosielski, Maciej Stawny

**Affiliations:** 1 Department of Pharmaceutical Chemistry, Poznan University of Medical Sciences, Rokietnicka 3, 60-806 Poznan, Poland; jczerniel@ump.edu.pl (J.C.); 87263@student.ump.edu.pl (J.D.); 2 Doctoral School, Poznan University of Medical Sciences, Bukowska 70, 60-812 Poznan, Poland

**Keywords:** flavonols, quercetin, fisetin, nanotechnology, drug delivery systems, gut microbiota, parenteral nutrition

## Abstract

Flavonols, representing a subclass of flavonoids, are an important group of polyphenols. Their activity is associated with a number of beneficial properties, including hepatoprotective, senolytic, neuroprotective, and anticancer properties. They are found abundantly in many fruits, vegetables, and plant products, but flavonols’ chemistry and structural properties result in their low bioavailability in vivo. In recent years, more and more studies have emerged that aim to increase the therapeutic potential of compounds belonging to this group, including by developing innovative nanoformulations. The present work focuses on the various steps, such as chemical analysis of the compounds, preformulation studies using drug delivery systems, preclinical studies, and finally clinical trials. Each of these elements is important not only for the innovation and efficacy of the therapy but most importantly for the patient’s health. There are also a limited number of studies assessing the population concentration of flavonols in the blood; therefore, this review presents an up-to-date survey of the most recent developments, using the most important compounds from the flavonol group.

## 1. Introduction

**Figure 1:**
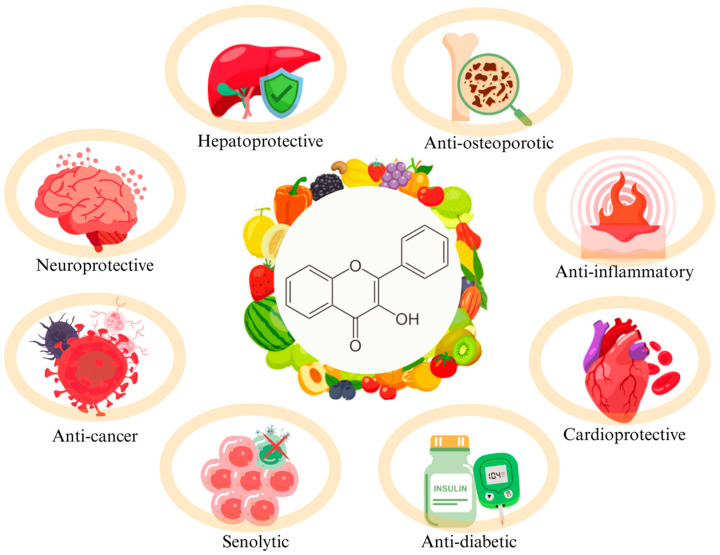
Flavonol properties.

Flavonols, as one of the main subclasses of flavonoids, are an important group of polyphenolic compounds with a broad spectrum of biological activity. Their synthesis takes place with the participation of light. They are concentrated mainly in plants’ outer and above-ground parts, so their presence is particularly visible in the peel and leaves of fruits and vegetables. They are rarely found in the parenchyma. Although this group of flavonoids is widely distributed in edible plants, their content usually remains low [1]. Structurally, they are characterized by the presence of a ketone and hydroxyl group on the C6-C3-C6 backbone, which determines their chemical and pharmacological properties [2]. The biological importance of flavonols is primarily due to their strong antioxidant properties, which enable them to neutralize free radicals and prevent oxidative stress, one of the main mechanisms leading to the development of civilization’s diseases. Due to their broad spectrum of activity and relatively low toxicity, flavonols are currently the subject of numerous preclinical and clinical studies as potential bioactive substances for adjuvant therapy as well as cancer prevention [3]. In recent years, compounds such as fisetin, quercetin, and kaempferol have been of particular interest to scientists, not only because of their potential to inhibit cancer cell proliferation, induce apoptosis, and modulate signaling pathways, but also in the context of their interaction with the gut microbiota and their potential for use in nutritional therapy. Moreover, more and more research focuses on the role of the gut microbiome in the metabolism of this subclass of flavonoids, which affects their bioavailability and results in the formation of secondary compounds with therapeutic potential [4,5,6]. The flavonols mentioned above are an extensively studied group in terms of chemical structure and biological activity, especially their anti-cancer and anti-inflammatory potential, which is associated with more work related to their use in preliminary research and clinical trials [7]. However, less-known flavonols, such as astragalin and myricetin, are also noteworthy. Despite preliminary studies indicating their unique biological properties, their pharmacological potential often remains untapped. This problem relates to unused phytochemical resources, and the authors of the articles emphasize the need for further research to expand the therapeutic spectrum of flavonols [8,9]. Epidemiological studies show that the regular consumption of flavonols is associated with a reduced risk of developing many chronic diseases, including cardiovascular diseases. An important role is also played by the development of therapeutic drug delivery systems, such as lipid–polymer hybrid nanoparticles, intravenous fat emulsions, or biopolymer complexes, which enable the effective delivery of active compounds, overcoming the limitations associated with low bioavailability [6,10,11]. According to bibliometric data from the Scopus database, the number of publications on the bioactivity of flavonols has more than quadrupled over the last decade [12]. Figure 1 shows the most important properties exhibited by flavonols.

## 2. Flavonol Chemistry

### 2.1. Classification

Polyphenols are a large group of secondary plant metabolites characterized by the presence of one or more aromatic rings with attached hydroxyl groups [13,14]. Within it, there are two main classes: flavonoids and non-flavonoids, such as phenolic acids, stilbenes, and lignans. The symbol C6-C3-C6 describes the basic structural arrangement of the first of the mentioned groups. The chemical core of flavonoids is based on a diphenyl-propane skeleton made of fifteen carbon atoms. It involves two aromatic rings connected by a three-carbon bridge, sometimes forming an additional, third heterocyclic system containing an oxygen atom [15,16]. Analyzing the different oxidation states and the position of the rings, the following subclasses are distinguished: flavonols, flavanones, flavan-3-ols, anthocyanins, flavones, and isoflavones [17].

**Figure 2:**
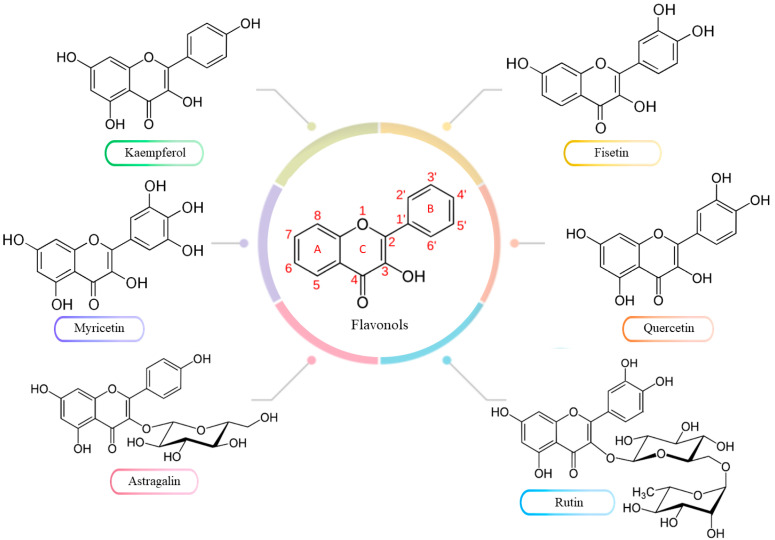
Structural formulas of the most important flavonols.

Flavonoids exhibit numerous beneficial therapeutic effects due to their anti-inflammatory and anti-aging properties, and they are also valued as nutraceuticals. Moreover, they can interact with various molecular and cellular pathways, including cell cycle regulation and apoptosis, making them promising candidates for treating gastrointestinal cancers [18]. Their main feature is high antioxidant activity, leading to the elimination of damage caused by free radicals and the alleviation of oxidative stress [19]. The most abundant subclass of flavonoids is flavonols, which display diverse methylation patterns, hydroxylation, and glycosylation patterns. They can occur in nature in the form of O- and C-glycosides, similar to related compounds such as flavones, isoflavones, coumarins, and isocoumarins, which are considered valuable natural products with documented biological activity [20,21,22]. The core structure of flavonols is typically 3-hydroxyflavone, also known as 3-hydroxy-2-phenylchromen-4-one (Figure 2). Due to their structural characteristics, flavonols are sometimes described as hydroxylated derivatives of certain flavones. They are widely present in the diet, particularly in fruits, green vegetables, beverages, and various medicinal plants and herbs [23,24].

### 2.2. Structure–Activity Relationships

A characteristic feature of the flavonol structural skeleton is the presence of a double bond between C2 and C3, a hydroxyl group at C3, and a ketone group at C4 [25,26]. This structural motif, common to all flavonols, gives this group its distinctive properties. It has been noted that significant antioxidant activity is associated with the presence of a free 3-OH group and the possibility of conjugation between the aromatic rings. Hirano et al. [27] investigated the antioxidant activity of various flavonoids. They found that the TEAC (Trolox Equivalent Antioxidant Capacity) of quercetin, which contains a hydroxyl group at the C3 position, was more than twice as high as that of luteolin, a flavone with a hydrogen atom at C3. These compounds differ only in the nature of the substituent at C3. Furthermore, all tested flavonols demonstrated stronger DPPH (2,2-diphenyl-1-picrylhydrazyl radical) scavenging activity compared to luteolin, confirming that free radical scavenging capacity is strongly influenced by the presence of a free C3-OH group [27,28].

Moreover, C3-OH substitution with a methyl or glycosyl group significantly reduces the activity of quercetin and kaempferol against β-carotene oxidation in linoleic acid in the DPPH assay [28]. Nevertheless, rutin, which is a derivative of quercetin formed by substitution of a phenolic group at the C3 position with a disaccharide (rutinose), shows only slightly weaker activity than quercetin in suppressing Fe(II)-induced MDA formation in liposomes [29] and ascorbic-acid-induced lipid peroxidation in rat mitochondria [30]. Discrepancies related to the antioxidant activity of rutin may be associated with the methodology of the studies conducted [30].

The free substituent C3-OH increases the stability of the flavonoid radical by affecting the spatial structure of the B ring, the heterocycle, and the A ring. The hydrogen bond between C3-OH and the hydroxyl groups in the B ring maintains the flavonol structure’s planarity, enabling conjugation, electron delocalization, and a corresponding increase in the stability of the flavonol phenoxyl radical. The intramolecular hydrogen bond with C3-OH is subject to particular enhancement in the presence of C3′, C4′-catechol, which explains the strong antioxidant activity of flavon-3-ols such as quercetin. Eliminating this hydrogen bond results in a slight twisting of the B-ring, thus worsening the electron delocalization capacity and consequently weakening the scavenging ability [31,32].

Nevertheless, the presence of C2-C3 unsaturation in conjugation with the C4 carbonyl group is essential. Although no consistent correlation has been shown between the presence of a C2-C3 double bond and antioxidant activity in methanolic DPPH solution [28], a comparative study between quercetin and taxifolin suggests that the presence of a C2-C3 double bond and a C4-carbonyl grouping distinguishes the better antioxidant [33]. Moreover, these flavonol-specific groups show lower IC_50_ values, indicating more potent antioxidant activity in the microsomal system compared to flavonoids with saturated heterocyclic bonds [34]. The presence of a double bond between the C2-C3 of the C-ring and the C4-carbonyl group has also been linked to the anticancer properties of flavonols by affecting topoisomerase I (topoI) and II (topoII) activity. Flavonols such as quercetin, fisetin, and myricetin strongly inhibit topoI activity. However, differences in their mechanism of action have been noted. Myricetin (with a slightly twisted B-ring due to the C5’-OH) acts as a covalent topoI poison stabilizing the enzyme–DNA complex, while for the planar quercetin, a DNA-intercalating action is proposed [35]. Inhibition of topoII by flavonols is associated with the formation of a planar pseudo ring involving a ketone group at the C4 position (C ring) and a C5-OH moiety (A ring) [26].

The phenolic characteristics of flavonols are derived from the attachment of one or more hydroxyl groups to their benzene rings, typically at positions C5 and C7 on ring A, as well as C3′, C4′, and C5′ on ring B. In addition to these structural features, the quantity and positioning of the hydroxyl groups play a critical role in determining the physicochemical properties and bioactivity of each flavonol [25]. The antioxidant activity of flavonols, resulting from preventing the formation of fasting reactive oxygen species (ROS) and/or facilitating their removal, is also closely related to the configuration and number of phenolic groups bound to the B ring. ROS stabilization occurs by transferring a hydrogen atom or electron from the phenolic groups, accompanied by a redox reaction and the formation of a stable flavonol radical [25,36].

The system characterized by the strongest ROS scavenging is related to the structure of C3’C4’ catechol in the B ring, which strongly enhances the inhibition of lipid peroxidation [37]. C3’C4’-catechol oxidation occurs with facilitated electron delocalization and leads to the formation of a stable ortho-semi-quinone radical [29,38]. The described catechol system is present, for example, in fisetin, quercetin, and its glycosidic derivative, rutin (Figure 1). Studying the effects of flavonols on topoI and topoII, it was noted that a phenolic group at C4’ is essential for the redox-independent poisoning of topoII. Phenolic groups at the C3’ and C5’ positions increase redox sensitivity, thus enabling it to act as a redox-dependent topoII poison [26]. According to the observations, the topo2 inhibitory properties of selected flavonols can be ranked starting from the strongest: quercetin > kaempferol > fisetin > myricetin. The specific structure of flavonols enables their oxidation to form 2–benzoyl–2-hydroxy-3(2H)-benzofuranones (BZFs) as primary metabolites, which are formed independently of the number and position of the phenolic groups of the B ring. Notably, a significant increase in antioxidant properties was observed upon conversion of flavonols to the corresponding BZFs relative to their precursors [25].

**Figure 3:**
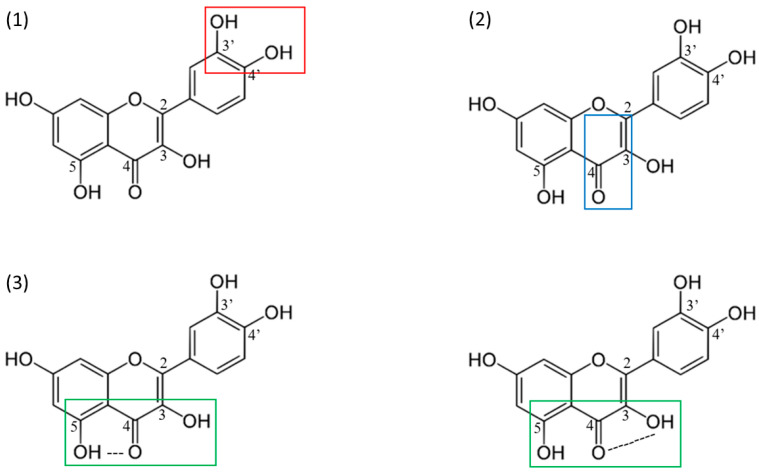
Structural groups for radical scavenging. (**1**) Electron delocalization is promoted by the presence of two hydroxyl groups at C3′ and C4′ in the B ring (indicated in the red box), forming a catechol structure; (**2**) the presence of a double bond at C2-C3, when conjugated with the C4-keto group (highlighted in blue), promotes the spread of electrons from the B ring; (**3**) the -OH groups at the C3 and C5 positions (surrounded by a green border) allow the formation of an intramolecular hydrogen bond with a carbonyl group.

In summary, the antioxidant activity of flavonols is influenced by three key factors: (1) the presence of a C3′, C4′-dihydroxy (catechol) structure in the B ring, which promotes electron delocalization; (2) the unsaturated bond between C2 and C3, which, when conjugated with the C4-keto group, enhances electron delocalization from the B ring; and (3) the hydroxyl groups at positions C3 and C5, which form an intramolecular hydrogen bond with the ketone group. The more of these factors that are present, the greater the antioxidant power of the flavonol. This is evidenced by quercetin, which fulfills all three factors mentioned above and demonstrates a very strong ability to scavenge free radicals (Figure 3) [39,40].

The precise role of various hydroxyl configurations may not yet be fully understood. Still, it is noteworthy that increasing the number of hydroxyl groups does not substantially affect the antioxidant activity associated with A-ring substitutions. Interestingly, when examining the B-ring, the arrangement of hydroxyl groups on the A-ring (except 5-OH) appears to have a limited impact on antioxidant effectiveness [30].

## 3. Therapeutic Drug Delivery Nanosystems

Flavonols exhibit a wide spectrum of biological activity, but their therapeutic potential is limited. This is mainly due to poor solubility in water, which limits intestinal penetration, and high lipophilicity, which promotes the penetration of cell membranes [41]. The presence of the gut microbiota and liver enzymes promotes the formation of aglycone forms, which are better absorbed than glycosides, as well as bioactive metabolites, such as phenolic acids, which exhibit independent pharmacological effects [42]. The mechanism of transport by enterocytes based on passive diffusion and proteins (e.g., OATP, MRP2) is also important [43,44]. Phase I metabolism may lead to oxidation and inactivation of the compound, while in phase II, as a result of glucuronidation and sulfurization, conjugated forms with different biological activity are formed [44,45]. Therefore, maintaining the therapeutic effect requires the regular and frequent administration of the agent.

The solution is a nanoparticle-based drug delivery system with a size range of 1 to 1000 nm. Together with the shape, it limits the behavior of nanostructures in vivo, including the efficiency of passive or active drug transport, tissue accumulation, toxicity, or elimination from the body [46,47]. Due to their large surface area, they can interact with solvents, which in turn leads to increased solubility of bioactive substances. The described properties introduced nanoparticles as an effective carrier in various drug delivery systems, improving their solubility, bioavailability, and bioefficiency [48].

Recent studies focusing on individual therapeutic systems using different flavonol compounds are presented in Table 1. We have divided the drug delivery nanosystems studied so far into those delivered by oral and parenteral routes, bypassing the gastrointestinal tract. To improve the readability of the presented data, we have separated the systems delivered through the skin as dermatological systems from the parenteral formulations group.

### 3.1. Parenteral Formulations

Parenteral administration of a substance involves the introduction of a drug or nutrient bypassing the gastrointestinal tract, most often intravenously, intramuscularly, subcutaneously or intrathecally. In recent years, as numerous studies have shown, this route of administration is particularly important in the case of compounds with poor water solubility, low bioavailability or requiring rapid action, such as fisetin, kaempferol, quercetin or myricetin [60,61,62]. Although the first articles on the use of flavonol-rutin in the form of infusion preparations date back to the 1950s, it is only in the last 15 years that there has been an intensive development of research in this area, which is related to the development of modern nanocarriers that allow efficient delivery of these structures. Selected studies on flavonols administered by various parenteral routes are shown in Table 2.

One groundbreaking example using fisetin is the work of Ragelle et al., in which the authors focused on developing a nanoemulsion targeting lung cancer cells with enhanced bioavailability. The system was characterized by a droplet size of 153 ± 2 nm, a negative zeta potential (−28.4 ± 0.6 mV), and stability for 30 days. The study was conducted with 8-week-old C57BL/6J mice with transplanted cancer cells. The experiment used a system containing 5 mg/mL fisetin, which contributed to increasing the accumulation of the drug in the tumor tissue and significantly reducing it compared to the control group. Significant differences were observed with intraperitoneal administration, which was characterized by a 24-fold increase in systemic exposure compared to the intravenous route [63].

In the following years, the fight against LLC was also discussed by developing a liposomal formulation containing fisetin, which allowed the delay of tumor growth. Increased efficacy of the therapy was achieved by the simultaneous inclusion of cyclophosphamide. The relative bioavailability of liposomes was 47-fold greater compared to the free compound [62]. The work on MCF-7 breast cancer treatment focused on optimizing the formulation of fisetin-based nanocochelates, unique supramolecular lipid structures that were characterized in terms of particle size, physicochemical stability and compound encapsulation capacity. In vitro studies have demonstrated improved antitumor activity and safety against blood cells while improving bioavailability with intraperitoneal administration [64]. Talaat et al. [65] were the first to develop a biocompatible phytosomal system in which fisetin was combined with soy phosphatidylcholine in the presence of cholesterol. PEGylated cholephytosomes were targeted for the treatment of breast cancer using the MDA–MD-231 cell model. To enhance the anti-tumor activity, the carriers were modified with hyaluronic acid, allowing them to be targeted by phosphatidylserine and CD-44 receptors. The formulations were characterized by high stability, compound complexation efficiency of 100% and particle size below 280 nm. Biochemical studies confirmed better cell internalization (targeting CD-44 receptors and phosphatidylserine), decreased cell viability (lower IC_50_), apoptosis induction and tumor growth inhibition. In addition, for the first time, this study described the effect of the compound and its nanocarriers on the downregulation of TGF-β1 and related ERK1/2, NF-κB expression in a subcutaneous Ehrlich-induced breast cancer model [65].

Nicoleti et al. [66] developed NLCs containing kaempferol. The aim of the study was to improve the bioavailability of the compound and evaluate its efficacy against glioblastoma multiforme. The formulations, obtained by high-pressure homogenization, were characterized by an average particle size of 120 nm, a zeta potential of −21 mV and controlled release for up to 48 h. In an in vitro model conducted on the U087MG line, a seven-fold higher cytotoxicity was observed compared to the free compound. The high encapsulation efficiency of flavonol (93%) determined a cellular uptake of 75%. A problem in the treatment of glioma is the difficulty of drug penetration across the blood–brain barrier (BBB), which limits the effectiveness of many substances. Due to the small size and lipid structure of NLCs, this system has the potential to cross the protective BBB, which is crucial in the treatment of central nervous system tumors [66].

Quercetin is another example of a compound from the flavonol group with broad therapeutic potential. Purnama et al. [67] focused on the compound’s ability to inhibit the formation of new blood vessels in tumor tissues by reducing the activity of the factor VEGF-A. Through this mechanism, it shows potential in the treatment of retinal diseases such as vasoproliferative retinopathies. Due to the low ocular bioavailability of quercetin, determined by the hydrophobicity of the compound, the team developed thermosensitive nanoemulsions consisting of Pluronic F127 and Pluronic F68 and hydroxypropylmethylcellulose for intravitreal injection. The formulations effectively inhibited the migration and angiogenesis of umbilical vein endothelial cells, also reducing the expression and levels of VEGF-A in retinal pigment epithelial cells [67]. Despite the growing interest in the therapeutic use of flavonoids, several factors still limit their parenteral administration [64]. Nevertheless, studies focusing on alternative routes of administration have also developed systems with high therapeutic potential for parenteral use, as demonstrated in the case of kaempferol and myricetin [68,69].

### 3.2. Dermatological Systems

Dermatology is a branch of medicine that focuses on the diagnosis, analysis, and treatment of diseases affecting the skin, hair, nails, and mucous membranes. Various formulations, such as hydrogels, transdermal patches, and aerosols, are used to manage these conditions, often allowing patients to avoid dermatologic surgery. These delivery systems are characterized by their ability to bypass first-pass metabolism and maintain elevated drug concentrations in the bloodstream. However, it should be noted that this route of administration may cause side effects, including skin irritation, such as allergic or irritant contact dermatitis (ICD). The use of nanocarriers in topical formulations enhances the penetration of bioactive substances into the skin and enables, among other benefits, their prolonged release [70,71]. They facilitate developments in the field of dermatology by contributing to non-invasive vaccination, modern diagnostics, and transdermal drug delivery. The main results of the conducted research are shown in Table 3.

To improve the dermatological potential of fisetin, Moolakkadath et al. [72] used a Box–Behnken design model to optimize glycerosome-type nanocarriers by modifying the content of glycerol (20–40%) and phosphatidylcholine (Lipoid S100) and the sonication time. The final formulation had a particle size of 138.8 ± 4.09 nm and an encapsulation efficiency of 86.41 ± 2.95%. The spherical encapsulated morphology was confirmed using a transmission electron microscope (TEM). In the last phase of the study, the permeability of fisetin through rat skin was evaluated using the Franz diffusion cell system, and the penetration depth of rhodamine B-labeled glycerosomes was analyzed by confocal microscopy. The final gel formulation was characterized by good texture, stability, and controlled, prolonged release of the drug, making it suitable for treating skin conditions such as inflammation and skin cancer [72].

NLCs are another example of a formulation with fisetin. Using a design of experiment (DoE) approach, Kumar et al. [73] developed a nanoformulation that had a high encapsulation efficiency (78.16 ± 1.58%), a particle diameter of 135.0 ± 5.5 nm, and stability for 60 days. Melanoma cell lines (A-375 and B16F10) were used for in vitro studies. Compared to the free compound, NLCs increase cellular uptake, reduce IC_50_ (~3.2-fold), and induce cell cycle arrest in G1/S phase and apoptosis through regulation of BAX and p53. In vivo studies have confirmed effective inhibition of tumor metastasis to the lung and liver, making fisetin-loaded NLCs a promising therapeutic system [73].

Hydrogels are also a commonly used formulation. Su et al. [74] developed such a hydrogel containing kaempferol, prepared with Pluronic F127 and deep eutectic solvents (DESs). The formulation was characterized by a controlled release profile of the drug (97.43 ± 5.37 μg/mL over 60 h) and the inhibition of ROS production due to cellular antioxidant activity. At a concentration of kaempferol of 250 μg/mL, the ability to scavenge DPPH, ABTS, and superoxide radicals above 90% was observed. In in vitro studies, the hydrogel was shown to inhibit human keratinocyte (HaCaT) cell proliferation without inducing cytotoxicity. In a model of imiquimod (IMQ)-induced psoriasis-like lesions in mice, hydrogel significantly reduced skin lesions, disease severity index, and the expression of inflammatory cytokines (TNF-α, IL-6, IL-17A) [74]. A study by Sutthammikorn et al. [75] proved the efficacy of an extract containing both kaempferol and quercetin in diabetic wound healing. Topical application in mice induced the expression of key growth factors, while an in vitro study confirmed the proliferation of fibroblasts, keratinocytes, and human endothelial cells [75].

To improve the therapeutic potential of myricetin, Lin et al. [76] developed a nanofiber system using an electrospinning technique, designed to increase the solubility of the compound and improve its physicochemical properties. Incorporation of the compound into hydroxypropyl-β-cyclodextrin (HPβCD)/polyvinylpyrrolidone K120 (PVP) scaffolds improved solubility in water by more than 2500 times compared to the pure substance (0.32 ± 0.16 µg/mL). For the optimal system, in which the ratio of Myricetin:HPβCD:PVP was 1:20:12, a size of 38.27 ± 1.02 nm was obtained. The study showed that the developed nanofibers could reduce the cytotoxicity of the compound in the HaCaT cell line by forming inclusion complexes. Moreover, in a model of a UVB-damaged HaCaT cell line, the formulations showed a photoprotective effect by reducing cell death and decreasing the excessive production of ROS [76].

### 3.3. Oral Formulations

Oral administration remains the preferred route for both systemic and local drug delivery. However, this route presents several challenges, including poor stability within the gastrointestinal tract, extensive first-pass metabolism, and the risk of systemic adverse effects [77]. The development of nanoparticle-based drug delivery systems makes it possible to increase the bioavailability and solubility of flavonols, reduce side effects, and prolong circulation time [78]. Selected studies on the oral administration of flavonols are included in Table 4.

Fisetin, which has a low bioavailability (44.1%), was successfully incorporated into PLGA nanoparticles. As a result of the research, formulations with an average particle size of 187.9 nm, an incorporation rate of 79.3%, and a zeta potential of 29.2 mV were obtained. Optimization was made using the DOE approach, which is gradually beginning to displace one-factor-at-a-time (OFAT) experiments. Such nanoformulations showed significantly increased fisetin solubility (three-fold), as well as improved intestinal permeability (4.9-fold increase in the Everted Gut SAC assay), indicating the potential for increased oral bioavailability [78].

Another nanoformulation that increases the therapeutic efficacy of the compound is lipid polymer hybrid nanoparticles (LPHNPs). Awadeen et al. [79] used ultrasonication and double emulsion W/O/W to develop chitosan-coated oral nanoformulations. The optimized system had a zeta potential of 30.16 ± 1.416 mV, which indicates the stability of the mucoadhesive strength (35.64 ± 0.55%). Fisetin in formulations showed significantly improved solubility (nine-fold) and controlled, prolonged release at different pH values. In vivo studies were performed in a model of L-arginine-induced acute pancreatitis (SAP) in rats. Biochemical markers of inflammation and organ damage (amylase, lipase, ALT, AST, creatinine, CRP) were significantly reduced after treatment with LPHNPs. The formulations reduced oxidative stress (MDA decrease, GSH increase) and inhibited the activity of NF-κB, TLR4 and the level of inflammatory cytokines (NLRP3, TNF-α, IL-1β, IL-6) [79].

To prepare the miricetin-loaded chitosan nanoparticles, the ionotropic gelation technique was used. The study focuses on the development of oral formulations targeting the treatment of type 2 diabetes mellitus (T2DM) as an alternative to the currently used injections that cause soreness and side effects. In vivo pharmacodynamic studies confirmed a reduction in pathological biomarkers and controlled weight gain. A reduction in pathological biomarkers was also observed, and histopathological studies showed no changes in major organs compared to the control group. The above study proved the safety of the nanoformulation with myricetin after oral administration [80].

Nanospheres (NSs) and nanocapsules (NCs) of zein (a protein found in corn) with entrapped quercetin were prepared to improve the oral bioavailability of the compound and its antihyperlipidemic effect. The nanocarriers showed a particle size of 225 to 255 nm, a negative zeta potential (below –40 mV) and an encapsulation efficiency of 82%. Scanning electron microscope (SEM) images confirmed the spherical shape and less stable structure in the nanocarriers compared to zein NSs, characterized by a hollow, cohesive structure. The in vivo model used Wistar rats, which were administered oral formulations at doses of 15 mg/kg. The relative bioavailability of NS was 57%, twice that of NC (26%). According to the general idea, NSs, classified as lipid forms, have greater potential in improving the bioavailability of lipophilic compounds, limiting their metabolism and promoting their absorption by the lymphatic system. In the case of the described study, the weaker properties of NC can be explained by their limited mechanical strength and faster passage through the gastrointestinal tract, proving that NSs are a better therapeutic option [81].

An example of a formulation with rutin is bilosomes (bilayer nanobubbles stabilized with bile salts). The aim of this study was to improve the stability and bioavailability of a compound from the flavonol group, as well as to evaluate their renal protective effects in a model of acute potassium dichromate-induced nephrotoxicity. A thin film hydration method was used to develop the nanocarriers. The size of the developed bilosomes ranged from 502.1 ± 36 to 665.1 ± 45 nm, which was significantly dependent on the concentration of sodium cholate. An in vitro release study showed a prolonged mechanism of rutin release from bilosomes compared to the free drug. The nanocarriers stimulated activation of the Akt/PI3K signaling pathway, which is crucial for protecting renal cells from apoptosis and oxidative stress. Histopathological studies were consistent with in vivo results and confirmed the protection of renal structure and reduction in damage after bilosome treatment. This confirms the validity of their consideration as a potential therapeutic system to improve the oral bioavailability and pharmacological activity of rutin [82].

Despite the preponderance of studies focusing on the positive aspects of oral flavonols, there are reports that question the validity of their application. Pihl et al. [83] examined the effects of fisetin, quercetin and rutin supplementation on ultraviolet radiation (UVR)-induced photocarcinogenesis in hairless mice. The experiment was conducted for a period of 9 months, in which the animals received 100 mg/kg of flavonols daily and a positive control of 600 mg/kg nicotinadmide. Oral application of fisetin and quercetin shortened the time of appearance of the first tumors and accelerated their growth, while rutin showed no significant changes. Analysis of the accumulation of compounds and their metabolites in the skin was carried out using mass spectrometry imaging (MSI). However, they were not detected, suggesting that the mechanisms of action are not fully elucidated and do not depend on their local accumulation. The study presented here shows for the first time that the oral administration of quercetin and fisetin can enhance the development of UV-induced skin cancers [83].

## 4. The Role of Flavonols in Gastrointestinal Dysfunctions and Prophylaxis

### 4.1. Flavonols Impact on the Gut Microbiome

In recent years, increasing attention has been paid to the potential impact of the gut microbiome on human health. Disturbances in the intestinal flora have been linked to the incidence of cardiovascular and neurodegenerative diseases and may also cause gastrointestinal dysfunction, including diarrhea and various inflammatory conditions of the intestines and stomach. The human microbiota comprises a large number of bacteria, viruses, and fungi. Among the predominant bacterial phyla present in the intestines of healthy individuals are *Firmicutes*, *Bacteroidetes*, *Proteobacteria*, *Fusobacteria*, and *Actinobacteria* [85]. Research has shown that the metabolism and bioavailability of flavonols, and consequently their biological activity, depend on the microorganisms that make up the gut microbiota. This relationship lies somewhere between commensalism and symbiosis: on the one hand, microorganisms facilitate the therapeutic effects of flavonols, while on the other, the microbiota benefits from the beneficial properties of polyphenolic compounds, which promote the growth and development of bacterial colonies [86].

Bacteria such as *Escherichia coli, Bifidobacterium, Eubacterium, Lactobacillus, Bacteroides*, and *Streptococcus*, which reside in the intestines and constitute the intestinal microbiome, are responsible for the biotransformation of flavonoids. This process leads to the formation of flavanol derivatives that also exhibit pharmacological activity. One notable example of flavanol biotransformation is the conversion of quercetin into quercetin-3-O-glucoside by *Bacteroides* species, specifically *B. fragilis, B. distasonis, B. ovatus*, and *B. thetaiotaomicron*. Quercetin-3-O-glucoside demonstrates improved bioavailability compared to quercetin itself while maintaining similar pharmacological properties. Both forms of this flavonol share the same mechanism of action, inhibiting the MAPK and NF-κB pathways, thereby exerting anti-inflammatory effects. The biotransformation of myricetin also involves the gut microbiome, primarily through methylation reactions. These methylation processes utilize substrates such as acetyl coenzyme A and result in the formation of mono- and di-methylated derivatives of myricetin [87,88].

The influence of flavanols on the gut microbiota is primarily mediated through the modulation of the composition of the intestinal flora. This process involves promoting the proliferation of beneficial bacterial species while simultaneously inhibiting pathogenic microorganisms. Such modulation contributes not only to the prevention and treatment of various diseases but also to the overall maintenance of intestinal homeostasis. Studies have demonstrated a positive correlation between flavanol administration and the growth of *Lactobacillus* and *Bifidobacterium* species, which are microorganisms associated with reduced obesity risk. At the same time, a reduction in the populations of *Clostridium* and *Staphylococcus aureus* has been observed, thereby lowering the potential for infections and decreasing the risk of inflammatory bowel disease (IBD) [6,89].

Another health-promoting effect of flavanols on the intestinal microbiota is their role in modulating the integrity of the intestinal barrier. This occurs through interactions with desmosomes, which function as selective barriers limiting the translocation of harmful pathogens and substances while maintaining the absorption of essential nutrients. By increasing the abundance of beneficial bacteria within the microbiome, flavanols enhance the regenerative capacity and regulatory function of the intestinal barrier. Preserving the integrity of this barrier is of paramount importance for maintaining overall health and preventing infections caused by pathogenic microorganisms [90,91].

Flavonols contribute to the increase in their immunomodulatory activity, which is associated with the protective effect on the intestinal barrier, by stimulating the production of beneficial bacteria for the intestinal microbiome. A greater number of beneficial microorganisms will result in a greater amount of immunoglobulin A (IgA) secretion from the bacteria that live in the intestines. The immunomodulatory mechanism of flavonols occurs with the help of IgA, which is designed to counteract the adhesion of pathogens in the intestines and maintain the balance of the intestinal flora. Both IgA and short-chain fatty acids, which function as signaling molecules in this case, affect the immune system by preventing the formation of local and systemic inflammatory conditions [6,89,92].

### 4.2. Flavonols and Parenteral Nutrition

Flavonols may serve as valuable adjuncts in pharmacotherapy, particularly in patients receiving parenteral nutrition, who are deprived of the natural bioactive compounds normally obtained from fruits and vegetables. Owing to their antioxidant, anti-inflammatory, and antineoplastic properties, flavonols could help prevent complications associated with parenteral nutrition, such as hyperglycemia, hypertriglyceridemia, inflammation, and liver dysfunction, including intestinal-failure-associated liver disease (IFALD) [92,93].

Compounds such as quercetin, kaempferol, myricetin, and fisetin have demonstrated beneficial effects on glucose and lipid metabolism, as well as hepatoprotective properties. Both flavonols and their metabolites exert antidiabetic activity by modulating key metabolic pathways. They regulate biochemical processes involved in glucose homeostasis, including the inhibition of glycogenolysis and gluconeogenesis, and enhance insulin secretion by pancreatic cells [25,94,95].

Quercetin, for example, exerts hypoglycemic effects by influencing multiple molecular targets. It modulates phosphoinositide 3-kinase (PI3K) expression, thereby improving insulin-mediated glucose uptake, and activates 5’AMP-activated protein kinase (AMPK), which suppresses enzymes such as glucose-6-phosphatase (G6Pase) and phosphoenolpyruvate carboxykinase (PEPCK), leading to reduced gluconeogenesis. Furthermore, quercetin promotes the translocation of GLUT4 transporters to muscle cell membranes, enhancing peripheral glucose utilization. Kaempferol exhibits a comparable mechanism of action, affecting PI3K, AMPK, and STAT3 signaling pathways, resulting in GLUT4 translocation and improved glucose clearance. Similarly, myricetin and fisetin act through these pathways, reinforcing the antidiabetic and anti-inflammatory effects of flavonols [96,97,98].

Taken together, these mechanisms highlight the potential of flavonols as supportive agents in parenteral nutrition, contributing to the prevention of metabolic complications and enhancing the overall effectiveness of pharmacotherapy [93,99,100,101,102].

Flavonols may also find applications in the management of various intestinal diseases. Through their anti-inflammatory properties and capacity to modulate the gut microbiota, flavonols hold promise in the treatment of inflammatory bowel diseases (IBDs), such as Crohn’s disease and ulcerative colitis. They may also be beneficial in irritable bowel syndrome (IBS) and as adjuncts in the therapy of colorectal cancer, including in combination with chemotherapy. The anti-inflammatory effects of flavonols are mediated by their ability to inhibit multiple pro-inflammatory proteins and signaling pathways. Consequently, they may offer a safer alternative to nonsteroidal anti-inflammatory drugs (NSAIDs), which can exacerbate gastrointestinal mucosal damage and worsen symptoms in patients with IBD [103].

Inflammatory bowel diseases (IBD) are characterized by chronic inflammation of the gastrointestinal tract. Although their exact etiology remains unclear, oxidative stress and the associated overproduction of reactive oxygen and nitrogen species (ROS and RNS) are thought to play a key role. Flavonols such as quercetin, kaempferol, myricetin, and fisetin have shown potential in limiting IBD progression due to their antioxidant and anti-inflammatory properties. Quercetin, a potent antioxidant, reduces the production of pro-inflammatory mediators, inhibits RNS, and suppresses NF-κB activity, leading to decreased expression of pro-inflammatory cytokines and a reduced inflammatory state. Kaempferol similarly inhibits NF-κB and downregulates LPS-induced TLR4 overexpression, as well as the production of TNF-α, IL-1β, and IL-6. Myricetin also targets the TNF-α and NF-κB pathways, exerting comparable anti-inflammatory effects. These findings suggest that flavonols may serve as adjuvant therapies for patients with IBD or in the prevention of disease development [95,104,105].

IFALD as a liver disease associated with intestinal failure may be caused by various etiological factors, one of which is parenteral nutrition. The long-term use of parenteral nutrition has a significant impact on the liver’s condition and can lead to pathological changes within it. Such degenerations as cholestasis, fibrogenesis, and fatty liver infiltration due to the accumulation of fat from the parenteral nutrition mixture may occur. Since the exact etiology of IFALD is not known, there are several theories that explain its occurrence. One of them states that IFALD is responsible for the inhibition of the farnesyl receptor (FXR), which regulates the synthesis of bile acids from cholesterol in the liver. Quercetin and kaempferol have been identified as FXR agonists, which means these flavonols have the ability to regulate bile and cholesterol metabolism, making them potentially good compounds that, as an addition to parenteral nutrition, can prevent IFALD. Inflammatory cytokines such as TNF, IL-6, and IL-1 cause a decrease in the number of transporters capable of carrying bile acids in the liver. This phenomenon causes the accumulation of bile acids in the liver, which can lead to cholestasis. The anti-inflammatory properties of quercetin, kaempferol, myricetin, and fisetin enable the use of these flavonols as hepatoprotective compounds that prevent the occurrence of IFALD during parenteral nutrition [106].

### 4.3. Flavonols Bioavailability

According to the Food and Drug Administration (FDA), bioavailability is defined as the rate and extent to which an active ingredient or compound is absorbed from a pharmaceutical product and becomes available at the site of action. The bioavailability of flavonoids varies depending on their structural class. Flavonols, including quercetin, kaempferol, myricetin, and fisetin, are known for their particularly low bioavailability [42]. However, their derivatives often show improved bioavailability while maintaining similar pharmacological activity to the parent compounds. Glycosylation can enhance the bioavailability and gastrointestinal absorption of hydrophobic flavonols but at the cost of reducing their antioxidant capacity. Conversely, administration of flavonols in the aglycone form may facilitate more rapid absorption, as aglycones can be directly taken up from the small intestine. In contrast, glycosides require biotransformation and metabolism by, for example, intestinal microbiota before they can enter systemic circulation. Glycoside forms of flavonols can be hydrolyzed to aglycones by lactase-phlorizin hydrolase (LPH) prior to absorption. Another route involves the sodium-dependent glucose transporter (SGLT1), which facilitates the uptake of glycosides into enterocytes, where they are subsequently hydrolyzed by cytosolic β-glucosidase to aglycones. These aglycones are then transported via the bloodstream to the liver, where they undergo phase I and II metabolism, including reactions such as methylation, glucuronidation, and sulfonation. The bioavailability of flavonols is influenced by numerous factors, including sex, age, ethnicity, health status, disease presence, and genetic background. Diet also plays a significant role, as flavonols are lipophilic compounds and their bioavailability can be increased by a high-fat diet [107,108].

Fisetin exhibits a broad range of pharmacological activities but has limited therapeutic potential due to its low bioavailability (44.1%), poor water solubility (10.45 μg/mL), and considerable lipophilicity (logP 3.2). Pharmacokinetic data indicate rapid absorption followed by phase II biotransformation to sulfates and glucuronides. Following intraperitoneal administration (223 mg/kg), a maximum plasma concentration of 2.53 μg/mL was reached within 15 min [48]. Similarly, kaempferol shows poor oral bioavailability (approximately 2%), dependent on sugar moieties, with some free aglycone detectable in blood and urine. Its hepatic and renal transport involves organic anion transporters and ATP-binding cassette proteins, although the precise absorption mechanism of its glucuronides remains unclear [107,109]. Oral administration of myricetin results in bioavailability between 9.6% and 9.7% at doses of 50–100 mg/kg. Increased dosage raises Cmax and AUC, suggesting passive diffusion, while its prolonged Tmax (6.4 h) reflects poor water solubility [110]. Quercetin has a bioavailability below 10%, attributed to its hydrophobic nature and low water solubility (0.17–7 μg/mL). The aglycone form is even less soluble than glycosides. Its bioavailability is further limited by efflux from enterocytes into the intestinal lumen. In vivo, quercetin undergoes sulfonation to form quercetin-5’,8-disulfonate, which improves water solubility and has demonstrated greater anticancer activity against colon and breast cancer cells (MCF-7) compared to quercetin itself [111,112].

## 5. Flavonols in the Human Body: Concentrations and Clinical Applications

Over the past few years, there have been more and more studies on population plasma levels of flavonols. Both the concentrations of substances from the diet and the compounds of conjugated metabolites (glucuronides and sulfates) are measured. An example of this is quercetin, which is one of the better-known flavonols. It has proven biological activity but is not detected in plasma unchanged after oral administration. Instead, its metabolites circulate in the body, which are characterized by limited in vitro activity. Current data indicate that quercetin undergoes an intensive metabolic transformation to methylated derivatives and derivatives conjugated to glucuronate and sulfate, of which only glucuronides can be hydrolyzed at the level of blood vessels, leading to local release of the aglycone form. The conjugation process can therefore be reversible, and in the context of vasodilation and hypotensive effects, the cycle of coupling and cleavage of metabolites seems to be a necessary mechanism. Glucuronide metabolites act as carriers of quercetin and its methylated derivatives, enabling their delivery to tissues, where the final active compound is free aglycone. Analyses conducted in Europe, North America and Asia have shown significant differences between populations, which may be related to different dietary structure, genetic predispositions and the gut microbiota affecting flavonoid metabolism. Importantly, blood flavonol concentrations are increasingly included as biomedical markers in clinical trials of cardiovascular, cancer, neurodegenerative and metabolic diseases [113,114,115,116].

### 5.1. Population Blood Levels of Flavonols

Blood concentrations of flavonols are a key indicator of their bioavailability and potential health impact. At the beginning of the 21st century, a cross-sectional study conducted in northern Japan examined flavonol intake among 115 women aged 29–78 years. The average daily intake of flavonoids, including myricetin, fisetin, kaempferol, and quercetin (which accounted for 56% of total intake), was 16.7 mg. Flavonoid content in food products was determined by high-performance liquid chromatography (HPLC) with UV detection following sample extraction and hydrolysis. Dietary data were correlated with blood biochemical parameters, including total cholesterol (TC), LDL-C, HDL-C, and triglycerides (TG), measured using enzymatic methods. Statistical analyses, controlling for age, BMI, and energy intake, showed a significant inverse correlation between flavonoid intake (particularly quercetin) and TC and LDL-C levels (*r* = −0.261 and *r* = −0.263; *p* < 0.01). The results showed that quercetin may have a beneficial effect on the lipid profile and partly explain the lower incidence of cardiovascular disease in the Asian population compared to the European population [117].

Similar research was carried out two years later in Europe. The study involved 48 German female students with an average intake of the flavonols quercetin and kaempferol of 17.9 mg/d (quercetin) and 4.7 mg/d (kaempferol). The concentration of compounds in the blood was determined by HPLC, and their average values were 22.9 nmol/L and 10.7 nmol/L, respectively. Analysis of intra-individual variability showed a large discrepancy in blood levels of flavonols, suggesting that the use of biomarkers alone may not be sufficient to accurately assess long-term consumption of these substances [118].

A study by Cao et al. [119], conducted in Asia, involved 92 students aged 20 to 28 who, as before, followed a customary diet. The mean intake of flavonoids was 13.58 mg/day for quercetin, 14.97 mg/day for kaempferol and 12.31 mg/day for isorhamnetin. In order to quantitatively analyze the levels of flavonols in plasma, the HPLC method was used again, and they were 80.23, 57.86, and 39.94 nmol/L, respectively. According to the results of the statistical analysis, flavonol intake had a significant correlation with blood concentrations, and the correlation coefficients ranged from 0.33 (isorhamnetin) to 0.51 (quercetin; *p* < 0.05) [119].

Popiolek-Kalisz and Fornal [120] evaluated dietary flavonol intake in patients with coronary artery disease (CAD) in adult Poles. To this end, a food frequency questionnaire (FFQ) was developed and validated, which included 140 products containing compounds such as quercetin, kaempferol, myricetin and isorhamnetin. The study involved 103 people, including 43 patients with CAD and 60 healthy controls. Intake of isorhamnetin was significantly lower in patients with CAD (mean 2.70 mg/day) compared to healthy participants (mean 5.72 mg/day), suggesting that it may have an important role in protection against CAD. Among other flavonols, no significant differences were observed between the study groups [120].

The FFQ was also used in a study by Sadeghi et al. [121], which assessed the relationship between flavonol intake and blood levels using HPLC. The study included 140 Iranian patients diagnosed with breast cancer who followed an Iranian diet (similar to the Mediterranean diet), rich in fruits and vegetables, which provided various amounts of flavonols, especially quercetin and kaempferol. For the aforementioned compounds, daily intake was 67.7 mg/day and 24.4 mg/day, respectively, as well as 4.3 mg/day for isorhamnetin. The average blood concentrations of quercetin, kaempferol and isorhamnetin were 102.5 nmol/L, 74.3 nmol/L and 897 nmol/L, respectively. The results of the analysis showed a significant correlation between the intake of flavonols and their blood concentrations, particularly for quercetin [121]. Available pharmacokinetic studies involving fisetin indicate that following its oral administration in healthy individuals, maximum blood concentrations (C_max_) typically reach values on the order of 1–2.5 µg/mL within the first 15–60 min after intake. However, in individuals who do not regularly supplement with flavonol in any way, fisetin levels are much lower, below the detection limit [122,123]. At higher doses (50 mg/kg), serum concentrations fluctuated, with maximum plasma concentrations of 2–3 μg/mL [124]. Pharmacokinetic analysis of quercetin (mainly in the form of the glycoside rutin) showed that after ingestion, flavonol undergoes phase I and II metabolism in the liver. After ingestion of 500 mg of the compound, its metabolites reach maximum blood concentrations (0.5–1 μM) within 0.5 to 1 h. Excretion occurs mainly in the urine over a 24 h period. After intravenous administration of the compound, the maximum plasma concentration can be up to 400 μmol/L, demonstrating that the bioavailability of quercetin is markedly higher with parenteral administration than after oral intake [107,125,126]. The process of kaempferol’s metabolism does not differ from that of quercetin. In the intestines, it is converted to the aglycone form, which is not always the case with quercetin. The level of kaempferol in the blood ranges from 0.1 to 0.5 μM, while its peak concentration is reached after 5–6 h. It should be noted, however, that kaempferol concentrations in the blood, as with all compounds, depend on the route and source of administration. After the consumption of tea containing 14.97 mg/day, it reaches an average plasma concentration of 16.69 ng/mL, and after the consumption of 27 mg of kaempferol with tea, the concentration is 15 ng/mL. In contrast, in other studies, populations with lower flavonol intake had a mean concentration of 10.7 ng/mL [127,128].

Dang et al. [129] studied the pharmacokinetics of myricetin in rats depending on the route of administration. They found that flavonol applied orally at a dose of 50 mg/kg reached a blood concentration of 1488.75 ng/mL after 6.4 h, while at 100 mg/kg, the concentration rose to 2,611.76 ng/mL after 5.2 h. When 0.5 mg/kg was administered intravenously, the C_max_ was 2232.16 ng/mL after just 1 min and then dropped to 7% of the initial level after about 2 h [129]. In another study, in which rats were given 100 and 300 mg/kg of myricetin for 6 weeks, a decrease in systolic blood pressure was observed. In addition, it contributed to lower plasma glucose, cholesterol, triglycerides and insulin levels in fructose-fed rats. This demonstrates the compound’s ability to improve the metabolic profile, which may be a result of reduced insulin resistance [130].

For astragalin, there are no human studies available to confirm its concentration. Studies in animal models have shown serum concentrations of 3.80 ± 0.38 ng/mL after 6 ± 2.24 min [131]. Population blood concentrations of flavonols vary according to diet, gender, health status and individual differences in biotransformation pathways, and their bioavailability and therapeutic efficacy may be limited by factors such as chemical form and interactions with other dietary components. While the available studies provide valuable information, further research is needed to better understand their fate in the system.

### 5.2. Clinical Trials Using Flavonols

Clinical trials are prospective biomedical studies that involve direct intervention, observation of patients or the use of cells or human tissue samples [132]. They are conducted in multiple phases or stages, and the results provide needed information to the FDA, based on which an evaluation of the benefits, safety and risks of a new drug or therapy is conducted [133]. Due to a number of beneficial properties and high therapeutic potential, flavonols are increasingly becoming the focus of clinical trials in the context of the prevention and treatment of chronic diseases.

Krishnakumar et al. [134] described a randomized, double-blind, crossover study that focused on the bioavailability and pharmacokinetics of fisetin. The compound was administered to 15 healthy volunteers between the ages of 22 and 55 with a BMI of 18–25 kg/m^2^, who were qualified from a 21-person group. Fisetin was applied in the form of a pure compound (UF) or a new hybrid hydrogel formulation (FF-20). Participants received a single dose of 1000 mg of FF-20 or UF, with a 10-day washout between doses. Blood samples were collected at various time points (0.5, 1, 2, 3, 5, 8, and 12 h after ingestion) to measure fisetin and its active metabolite, geraldol. Pharmacokinetic analysis showed better bioavailability (26.9 times greater area under the curve-AUC_0–12 h_; *p* < 0.0001) and better absorption (23.9-fold increase in C_max_; *p* < 0.0001) in those taking the oral formulation. The parameters and changes mentioned were analyzed using ANOVA with Dunnett’s test. Differences with *p* < 0.05 were deemed significant. No side effects were reported during the experiment, allowing the use of encapsulated fisetin as a modern therapeutic option [134].

In a medical experiment described by Akiyama et al. [135], the safety of excessive administration of KMP was investigated using a randomized, placebo-controlled, double-blind study. Gender, age, body mass index (BMI) and urinary KMP excretion levels, assessed at screening, served as stratification variables. Using stratified block randomization, participants were randomly assigned to the supplement or placebo group. Of the 95 volunteers participating in the initial study, 48 healthy adults (24 in the KMP group and 24 in the placebo group) were enrolled and given 50 mg of KMP or a capsule containing cornstarch-based powder for 4 weeks. This was followed by a 2-week observation period. The dosage of flavonol was five times the average dietary intake of the compound. To evaluate the safety of aglycone, general toxicity, hematological and biochemical blood parameters, urine quality, and adverse events were analyzed. Continuous variables were analyzed using a linear mixed effects model with treatment and time as fixed effects and participants as random effects. Missing data were not imputed. Categorical data were compared using Fisher’s exact test. Analysis of the results showed no abnormal changes in markers of renal function, such as creatinine and blood urea nitrogen. However, statistically significant differences were observed between the study groups in MCHC, direct bilirubin, γ-GTP, AST, ALT, Na, P and urine pH values. Their values were within normal limits, making them not clinically significant. Despite the favorable results, the study had a small sample and did not include the participation of people with comorbidities, children and pregnant women, which are limitations of the experiment. In the case of healthy adults aged 20 to 79 years, the safety of high doses of flavonol was confirmed, providing a starting point for the design of further clinical trials involving specific patient groups [135].

A randomized, controlled, single-blind study using myricetin was initiated in 2023. It reduced blood ethanol concentrations by activating ADH. Inclusion criteria included healthy subjects, aged 18–35, non-smokers, with no history of alcohol abuse. Ultimately, 20 subjects (13 males and 7 females) with a mean age of 21.4 ± 2.46 years participated in the study. The experiment was designed to evaluate the effectiveness of a new supplement, in which flavonol was one of the ingredients, in lowering blood alcohol levels and reducing biomarkers of oxidative stress. During the week-long study, blood, urine and saliva samples were collected to measure levels of alcohol, EtG (a marker of alcohol consumption), ROS, total oxidative capacity (TAC), coenzyme Q10, 8-isoprostastane, NO metabolites, neopterin, uric acid and thiol redox status. As a result, a 33% decrease in ethanol concentrations 2 h after supplement ingestion was observed compared to the placebo group, as well as lower ROS fluctuations. In addition, a 9–12% increase in TAC was observed, which was significantly correlated with EtG—those with higher antioxidant capacity excreted more EtG. Statistical analysis included a series of tests, such as Kolmogorov–Smirnov, to assess the normality of distribution, as well as ANOVA and Spearman correlations. The results confirmed the supplement’s effectiveness in faster alcohol metabolism and reduction of oxidative stress, as well as a statistically significant ethanol-reducing effect [136].

Among flavanols, quercetin is the most frequently described compound, including in the context of clinical trials. The study was prospective, single-center, open-label, and included phase 1 (dose-finding study) and an expanded phase with additional correlative analysis. The goal was to examine the safety, tolerance, pharmacokinetics, and preliminary efficacy of quercetin—a natural antioxidant—in children and young adults with Fanconi anemia (FA). The aforementioned condition is a rare genetic disorder associated with a DNA repair defect that leads to bone marrow failure (BMF) and an increased risk of cancer. One of the pathophysiological mechanisms of FA is the elevated level of ROS, which damages the bone marrow stem cells. In a group of 12 patients in the phase 1 cohort, a reduction in ROS in peripheral blood was observed in 58% of those treated after 4 months and in 78% after a year. In the expanded phase, which included 18 people, the greatest reduction was achieved at the maximum dose (4000 mg/day). A total of 21 patients treated with the target dose (the so-called analysis cohort) were analyzed. Statistical significance results indicated, among other things, a reduction in ROS in peripheral blood after 1 year (−75% in phase 1; *p* = 0.04), cohort analysis after 6 months (−25%; *p* = 0.67), and cohort analysis after 1 year (−18%; *p* = 0.06). In addition, there was an increase in ANC (neutrophil count) at 6 months (*p* = 0.01) and a decrease in hemoglobin levels at 12 months (*p* < 0.001). The study presented is the first analysis of the function of the flavonol with antioxidant properties in treating genetic disease. The observed biological benefits include a reduction in ROS, with possible positive hematological effects in some patients. However, further phases of studies are needed to assess the efficacy of the therapy clearly [137].

Bazyar et al. [138] described a double-blind, randomized clinical trial with a placebo group. The experiment aimed to evaluate the effects of rutin supplementation on blood pressure parameters, antioxidant enzyme levels, and quality of life (QOL) in patients with T2DM. A total of 50 subjects (25 in each group) were enrolled in the study. Using allocation concealment, randomization was performed in a block design (blocks of six with four codes). The 3-month follow-up of the study group showed significant reductions in systolic blood pressure (–8.60 ± 9.32; *p* < 0.001), diastolic blood pressure (–4.20 ± 5.78; *p* = 0.001), heart rate (–5.36 ± 5.67; *p* < 0.001), mean arterial pressure (–5.64 ± 6.02; *p* < 0.001) and pulse pressure (–4.32 ± 8.27; *p* = 0.007). At the same time, a significant increase in the levels of antioxidant enzymes superoxide dismutase and glutathione peroxidase was observed. The improvement in QOL was mainly related to mental and emotional aspects. Intracellular comparisons were made using the Student’s *t*-test for dependent samples or the Wilcoxon test. To evaluate intergroup differences, Student’s *t*-test for independent samples, the Mann–Whitney test was used. The above study confirmed the beneficial effect of rutin in patients with T2DM, and the lack of observed side effects allows its use in clinical practice [138]. Table 5 supplements the above text, considering additional clinical studies from recent years, for the selected compounds from the flavonol group.

### 5.3. Opportunities and Challenges Affecting Therapy Transfer

Translational research, also known as translational medicine, is the process by which the results of laboratory experiments can be implemented into clinical trials. Their main goal is to develop new treatment methods, devices, medical procedures, diagnostics, and prevention [147,148]. The presented actions aim to improve the comfort of patients’ lives by increasing the effectiveness of therapy. The National Center for Advancing Translational Sciences (NCATS), operating under the U.S. National Institutes of Health (NIH), is the organization responsible for the development of the new discipline and coordinating the stages between basic research and clinical research [149,150].

**Figure 4:**
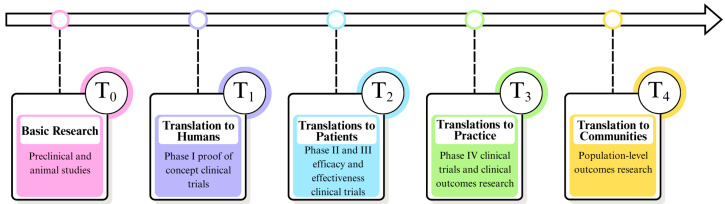
The translational research spectrum (interpretations in the text).

As shown in Figure 4, translation research occurs along a continuous scientific innovation process, consisting of five phases (T_0_ to T_4_). In practice, operational stages are characterized by many feedback loops and interrelated phases, which require continuous data collection, analysis, and dissemination of results [150,151].

Phase T_0_ involves basic research aimed at understanding cellular mechanisms and their relationship to the development of diseases, which enables the identification of potential therapeutic targets and the development of new treatment strategies, including innovative therapeutic molecules. In the next stage (T_1_), the first phase of clinical trials is conducted. Their main goal is to assess safety, confirm the mechanism of action, and perform preliminary verification of therapeutic assumptions. Phase T_2_ includes phases II and III of clinical trials. In randomized studies, the effectiveness of therapy is evaluated in groups of patients who represent a given disease. These studies often include control groups to ensure the reliability of the results. In phase T_3_, clinical trials of phase IV are conducted, which focus on improving the application of therapy in everyday clinical practice. The final stage (T_4_), on the other hand, involves population-level studies and comparative effectiveness analyses, which are designed to assess the final therapeutic value and cost-effectiveness of a given intervention in comparison to other available treatment methods [150,151,152].

Using animal models in translational research helps better understand diseases, diagnostic methods, and potential therapies, enabling in vivo research on safety and efficacy before testing humans. However, the genetic, anatomical, and physiological differences often make these studies’ validity questionable. Their inseparable part is ethical issues, and special attention is paid to the 3R principles—reduction, refinement, and replacement of animal use. They are designed to ensure that the tests are as humane and effective as possible. Alternatives such as in vitro models, organoids, and computational simulations offer ethical solutions, but animal models are still necessary in research on complex biological and disease processes, such as cancer, vaccine development, and tissue regeneration [147,153].

Many published biomedical research results are misleading or difficult to replicate. The reason for this may be differences in methods, such as the type of coating on the test tubes, the temperature of the cell culture, or the composition of the substrate. In addition, the key issue is the proper design of the experiment in preclinical in vitro and in vivo studies on animals. As a result, systematic errors may occur, which make it impossible to reproduce the process reliably and lead to incorrect conclusions [150,154].

Another key aspect concerns the differences between species, which are noticeable in anatomical structure and physiological characteristics, among other things. An example is a study that compared the digestive systems of humans and albino rats. They exhibit numerous similarities, such as the presence of the oral cavity, throat, esophagus, stomach, small intestine, and large intestine, but also significant differences resulting from different nutritional needs and environmental adaptations. Both species have salivary glands that synthesize secretory immunoglobulins (IgA), which play a role in the local immune response. However, there are significant differences in the structure of the stomach. In humans, there is a single-chamber stomach; in rats, there is a double-chamber stomach, with a forestomach designed for bacterial digestion. The rat’s small intestine is much shorter than the human one, and the cecum is more developed and distinguished by its funnel-like shape, reflecting a fiber-rich diet. Additionally, humans have an appendix, and their mouths contain tonsils that serve an immune function. Despite differences in size and some structural features, the general anatomical organization of the digestive systems of both species is very similar. However, they may be significant when using rats as models in experimental research focusing on the alimentary canal [155].

In literature, there are several examples that show the influence of a given factor on the results of research or the characteristics of the experimental model. One of them is the C57BL/6J strain, which allows for the study of multi-gene factors related to diet-induced obesity; however, the degree of weight gain in this model may show significant differences depending on the research conducted. The variations that occur are a result of the role of the gut microbiome and thermoregulation, as well as the need for animals to adapt to being raised in new conditions. However, they may incorrectly lead to the perception of C57BL/6J mice as a discordant model of diet-induced obesity [156,157].

The differences that occur may have a potential impact on the toxicological assessment. It is worth adding that both flavonoids and the flavonols that belong to them, in addition to a number of beneficial effects, can cause potential side effects. In the context of carcinogenicity and mutagenicity, quercetin, genistein, and daidzein exhibit two-phase effects. At low doses, they can promote the growth of cancer cells (MCF-7, T47D), while at high doses, they have a cytostatic effect [158]. Under conditions of iodine deficiency, quercetin and soy isoflavones disrupt thyroid hormone metabolism by inhibiting thyroid peroxidase (TPO) activity and binding to TTR proteins [159]. Some biochemical changes in liver function have been observed in the case of kaempferol, such as increased transaminase levels (including AST and ALT), which may indicate liver cell damage. Additionally, it caused an increase in triglyceride and cholesterol levels, but these changes were within the normal range [160]. Scientific reports analyzing fisetin and rutin have proven their safety, even in high doses. Experiments showed no signs of toxicity, and the highest concentrations administered, reaching up to 2 g/kg, did not cause changes in the structure of internal organs, such as the heart, liver, or kidneys. A similar situation occurs with the second of the flavonols, rutin. Its use improved the activity of liver enzymes (such as AST and ALT) and increased antioxidant indices, such as SOD, CAT, and GPx, in animal models. In the nephrotoxicity context, rutin reduces oxidative stress, prevents inflammation, and alleviates apoptosis (cell death), which are key mechanisms of drug-induced toxicity [161,162].

In summary, the limitations that translational sciences must face are caused by a number of components, such as interspecies differences, designing experiments at the preliminary research stage, and the toxicological profiles of compounds. Therefore, actions are necessary to bridge the translation gap between data on the effectiveness of therapy in animals and data on its application to humans. An example of a solution is phenotyping patients, which involves using various imaging techniques, molecular profiling, and clinical data. The desired data is collected continuously to define human diseases better. This approach requires access to patient samples and consideration of ethical issues. Deep phenotyping combines retrospective and prospective molecular data and other information, including tissue histopathology, with electronic health records (EHRs), which may enable the discovery of new molecular associations with diseases and their subtypes [163,164].

Another commonly used approach is the Framework to Identify Models of Disease (FIMD), a tool for systematically evaluating and comparing animal models. It covers eight key areas: epidemiology, symptomatology, natural history of the disease, genetics, biochemistry, etiology, histology, pharmacology, and endpoints. This tool allows the user to create radar charts that help compare disease models in terms of their similarity to human diseases. This allows models to be better adapted to specific research goals, increasing the reliability of the results [165].

Among the tools, there are also the Investigator’s Brochure and IB-derisk. Its purpose is to evaluate the scope of pharmacological activity of drugs obtained in preclinical studies in the context of clinical data. This allows for comparing pharmacokinetic and pharmacodynamic results obtained in animals with human data, which can help better select an animal model at the early stage of clinical trials [166].

## 6. Conclusions and Future Perspectives

Flavonols are among the secondary plant metabolites that accumulate in the skin and leaves of fruits and vegetables. Due to their broad therapeutic potential, compounds such as fisetin, quercetin, and kaempferol are being increasingly analyzed and used in clinical practice. This involves the use of drug delivery nanosystems and the selection of an appropriate route of administration, which significantly increases the bioavailability and stability of flavonols, thereby enabling their targeted delivery. The above review focuses on the importance of the flavonoid subgroup in the context of the various stages of research aimed at introducing innovative and safe therapies, confirmed by clinical trials. All the stages described focus on improving the bioavailability of the compounds, which is a major limitation that prevents their full potential. The presented articles highlight the important role of flavonols as bioactive compounds that can support the integrity of the intestinal barrier and modify the microbiota through individualized nutritional strategies.

Recent medical trends focus on the implementation of artificial intelligence, which can significantly contribute to the analysis of the mechanisms of action of flavonols, their derivation, and the design of novel drug delivery nanosystems based on deep quantitative structure–activity relationship (QSAR) and generative modeling [167,168,169].

Lopes et al. [167], conducted a comprehensive study aimed at implementing artificial intelligence (AI) to predict the antiviral activity of flavonoids against the respiratory syncytial virus (hRSV). The aforementioned pathogen is responsible for lower respiratory tract infections in infants and young children, resulting in millions of hospitalizations annually. Currently, only two drugs are available (Palivizumab and Ribavirin), but both have limitations such as cost, toxicity, or effectiveness. In the face of these challenges, the authors decided to use artificial intelligence algorithms combining artificial neural networks (ANNs) with genetic algorithms (GAs) to develop a model capable of classifying flavonoids as active or inactive against hRSV. The models were trained based on biological data (from in vitro research) and physicochemical properties of flavonoids, sourced from databases such as PubChem and Open Babel. Data were divided into three groups: empirical, theoretical, and combined, and each was used to train, validate, and test nine AI models. Built neural networks achieved very high prediction accuracy—up to 99% for theoretical data and over 83% for combined data. The models also passed the so-called “blind test,” in which 489 flavonoids whose activity against hRSV had not been previously studied experimentally were analyzed. Based on 1956 data sets, this analysis allowed for the selection of 10 flavonoids deemed most promising, according to the predictions of at least seven of the nine AI models. Included were flavones, isoflavones, and flavanes, which showed the greatest antiviral potential in post-treatment tests. AI can be extended to other compounds and viruses, significantly contributing to revolutionizing the initial stages of drug discovery [167].

In the work of Liu et al. [168], a modern approach to the extraction of flavonoids using artificial intelligence methods and ultrasonic technology is presented. In order to optimize the process efficiency, the RSM (Response Surface Methodology) methodology and appropriate AI models were used. As part of the experiments, the influence of four main factors was analyzed: extraction time, ethanol concentration, material-to-liquid ratio, and ultrasonic power. It was found that the ratio of material to liquid and the device’s power had the greatest impact on the extraction efficiency. The optimal conditions are 30 min, 60% ethanol, 1:10 ratio, and 120 W power. The presented study confirmed that combining AI and classical methods yields precise predictive models for the extraction of biologically active compounds [168].

Similar research, which also used AI tools to optimize the extraction process, was conducted a year later by Chu et al. [170]. Analyses by SEM and FTIR confirmed numerous perforations and damage to the cell walls, which facilitates the release of compounds and the stability of functional groups. In addition, the extracts showed strong antioxidant activity in vitro. This is another example of a study that confirms that AI integration significantly improves the prediction and optimization of process parameters [170].

The article by Yordi et al. [171] presents an interdisciplinary study that uses AI and machine learning (ML) methods to predict the total antioxidant capacity (measured as ORAC) of food products based on their flavonoid content. The study created a unique database based on existing USDA resources and used the chemoinformatics approach TOPSMODE to extract topological molecular descriptors of flavonoids. Four ML algorithms—Random Forest, Support Vector Machine, k-Nearest Neighbors, and Multi-Layer Perceptron—were then used to model and predict ORAC values. The analysis showed that the Random Forest model achieved the highest prediction accuracy, while the MLP had the lowest accuracy. The most important factor affecting the antioxidant value of food turned out to be the total polyphenol content (TPexp), not the amount of a specific flavonoid. The structural properties related to the hydrophobicity of the molecules were also of high value. This study shows that artificial intelligence can be an effective tool in dietetics and food science, enabling quick and accurate prediction of the bioactive properties of ingredients based on chemical data without the need for costly laboratory analysis [171].

Flavonols, as versatile bioactive compounds, remain the subject of intense research, and their growing importance in the prevention and treatment of civilization’s diseases makes them an extremely promising direction for future therapeutic and nutraceutical strategies.

## Figures and Tables

**Table 1:** Recent studies focusing on individual therapeutic systems using different flavonol compounds.

Flavonol	Administration Route	Formulation	Experimental Model	Disease	Conclusions	Ref.
Astragalin	oral	Polymeric nanocarriers	In Vitro study: − In Vivo study: Wistar albino rats	CCl_4_-induced liver injury	↑ hepatoprotective effect; ↓ markers of liver damage (SGPT, SGOT); ↑ bioavailability	[49]
Fisetin	Intraperitoneal (i.p.) oral	β-Cyclodextrin Nanosponges	In Vitro study: MDA-MB-231 In Vivo study: Female Wistar rats	Breast cancer	↑ cytotoxicity (↓ IC_50_); ↑ apoptosis (lactoferrin-coated); ↓ cell migration and ↓ tumor growth; cell cycle regulation (↓ cyclin D1 and Bcl-2, ↑ Bax)	[50]
	i.v.	Nanocrystals	In Vitro study: 3LL cancer cells; EA.hy926 endothelial cells In Vivo study: −	Lung cancer	↑ cytotoxicity; apoptosis induction; ↓ angiogenesis; change in cell morphology	[51]
Kaempferol	i.v. oral	Nanosuspension	In Vitro study: 4T1; U251; HepG2; SGC-7901 In Vivo study: Balb/c mice with 4T1 cells	Breast cancer	↑ cytotoxicity; ↓ migration of tumor cells (4T1, U251); ↑ apoptosis induction (↑ ROS production); ↑ internalization and accumulation in the tumor (EPR effect)	[52]
	topical	Platelet-derived extracellular vesicles	In Vitro study: HUVEC In Vivo study: C57BL/6J mice	Corneal Neovascularization	active internalization; ↓ migration of cells; ↑ formation of vascular structures; ↓ expression of inflammatory and angiogenic markers; ↑ retention within the eye; ↑ bioavailability and ↓ toxicity	[53]
	intranasal	Mucoadhesive nanoemulsion	In Vitro study: C6 glioma cells In Vitro study: Wistar rats	Glioma	↑ cytotoxicity (↓ IC_50_); ↑ apoptosis induction; ↑ intracellular uptake; biocompatibility with nasal mucosa	[54]
Myricetin	inhalation route	Solid lipid nanoparticles; Phospholipid complex	In Vitro study: A549; In Vitro study: -	Lung carcinoma	↑ cytotoxicity (↓ IC_50_); ↑ cellular uptake; ↑ activity in the MTT test conducted in an environment with pH = 6.6	[55]
Quercetin	topical	Quercetin-loaded mesoporous nano-delivery system	In Vitro study: PDLSCs RAW264.7 In Vitro study: rat alveolar bone defect model	Periodontitis	↑ osteo- and angiogenesis (OPN, CD31) ↓ expression of pro-inflammatory cytokinosteo- and angiogenesis (IL-6, TNF-α) in PDLSCs, (↓inflammation); ↓ levels of inflammatory genes and proteins (IL-1β, IL-6, iNOS, TNF-α) in RAW264.7 ↑ bone regeneration (↑ BV/TV)	[56]
	intra-articular	Thermosensitive Hydrogel	In Vitro study: chondrocyty; In Vitro study: Sprague-Dawley rats	Post-traumatic osteoarthritis	– cytotoxicity; – effects on cytotoxicity and proliferation; ↓ joint pain; ↓ cartilage degradation; – effect on synovitis	[57]
Rutin	oral	Liposomes	In Vitro study: adipocyty; In Vitro study: high-fat diet (HFD)-induced obese mouse model	Obesity	↑ cellular uptake; ↑ solubility; ↓ body weight (↓ expression of proteins associated with fat synthesis) ↑ expression of beige proteins associated with adipogenesis	[58]
	Oral	SNEDDS	In Vitro study: - In Vitro study: Sprague-Dawley rats; ICR mice	Thrombosis	↓ thrombus formation (↑ time to occlusion); inhibition of the enzyme PDI (important in the initiation of coagulation); –organ toxicity	[59]

↑—Increase of the specified parameter; ↓—decrease of the specified parameter.

**Table 2:** Recent studies about flavonols in parenteral administration.

Flavonol	Administration Route	Formulation	Against	Conclusions	Ref.
Fisetin	Intravenous Intraperitoneal	Liposomes	Lewis lung carcinoma (LLC)-bearing mice	After i.p. administration, a 47-fold increase in relative bioavailability compared to free fisetin. Improved antitumor activity. Improved tumor growth retardation with co-treatment with cyclophosphamide.	[62]
Fisetin	Intravenous Intraperitoneal	Nanoemulsion	LLC-bearing mice	After intraperitoneal administration, a 24-fold increase in fisetin relative bioavailability, compared to free fisetin. Improvement of the antitumor activity of the fisetin nanoemulsion compared to free fisetin.	[63]
Fisetin	Intraperitoneal	Nanocochelates	Human breast cancer MCF-7	A 1.3-fold improvement in vitro anticancer activity towards human breast cancer MCF-7 cells was observed. A 141-fold higher relative bioavailability in mice with low tissue distribution.	[64]
Fisetin	Intravenous	Cholephytosomes modified or not with hyaluronic acid	Human breast cancer cell line (MDA-MB-231) Ehrlich ascites carcinoma cells	About 10- and 3.5-fold inhibition in IC_50_ of modified vesicles compared with free fisetin and conventional fisetin-phospholipid complex, respectively. Comparable cytotoxicity that is significantly surpassing free drug cytotoxicity. TGF-β1 and its non-canonical related signaling pathway, ERK1/2, NF-κB and MMP-9, were involved in tumorigenesis suppression.	[65]
Kaempferol	Not define delivery system to the cancer cell in central nervous system	Nanostructured lipid carriers (NLC)	Glioblastoma multiforme human brain cancer	Increased kaempferol cytotoxicity in the U-87MG cell line. Promoted cellular uptake at 75%, confirming enhanced cytotoxicity in U-87MG cells.	[66]
Quercetin	Intravitreal	Nanoemulgel	Vascular endothelial growth factor-A (VEGF-A) inducing neovascularization from the retinal pigment endothelial cells	Inhibited migration and tube formation of human umbilical vein endothelial cells. Inhibition of VEGF-A gene expression and VEGF-A protein levels in nascent retinal pigment epithelial cells under hypoxic conditions.	[67]
Kaempferol	Ophthalmic drops	PVP nanocomplexes	Human corneal epithelial cells In vivo inflammation eye model Alkali burn injury on the central cornea in mice.	Significant improvement in in vitro parallel artificial membrane permeability, in vitro cellular uptake, and ex vivo corneal permeation of kaempferol. Improvement in ocular absorption in vivo test. Improvement in in vitro antioxidant activity and in vivo anti-inflammatory activity. Improvement in the treatment efficacy of corneal alkali burns.	[68]
Myricetin	Intravenous	Solution (dissolution in dimethylsulfoxide and dilution in 0.9% NaCl)	Excitability of nociceptive sensory neurons in vivo	The suppressive effects continued for about 20 min. An acute, intravenous administration reduces the SpVc nociceptive transmission, likely through the inhibition of the CaV channels and by activating the Kv channels.	[69]

**Table 3:** Recent studies on topically administered flavonols.

Flavonol	Formulation	Aim of Study	Conclusions	Ref.
Fisetin	Glycerosomes converted into a Carbopol^®^ gel	Development and optimization of glycerosomes.	The prepared fisetin-loaded glycerosomes gel was suitable for dermal application.	[72]
Fisetin	NLCs	Development of fisetin-loaded NLCs for better efficacy against metastatic melanoma.	Inhibition of melanoma-associated metastasis in the lungs and liver was improved by 5.9-fold and 10.7-fold, respectively. Fisetin-loaded NLCs as an effective tool against melanoma.	[73]
Kaempferol	hydrogel	Development of kemferol hydrogel to improve efficacy in a mouse model of psoriasis-like lesions.	Effective inhibition of HaCaT cell proliferation without causing significant cytotoxicity. Reduced psoriasis area and severity index, improved IMQ-induced histopathology and reduced expression of pro-inflammatory cytokines in skin tissue.	[74]
*Gynura procumbens* extract containing kaempferol and quercetin	*Gynura procumbens* crude extract mixed with Vaseline	Assessment of *Gynura procumbens* on wound healing in the diabetic milieu.	Accelerated wound healing and induced angiogenin expression, epidermal growth factor, fibroblast growth factor, transforming growth factor, and vascular endothelial growth factor. Promotion of vascular formation in the diabetic mice.	[75]
Myricetin	Nanofiber system of hydroxypropyl-β-cyclodextrin or polyvinylpyrrolidone K120-loaded with myricetin	Enhance the water solubility and skin penetration of myricetin, antioxidant and photoprotective activity.	Increase in water solubility and permeability. Reduction of cytotoxicity in HaCaT cell lines. Better antioxidant and photoprotective activity	[76]

**Table 4:** Recent studies on orally administered flavonols.

Flavonol	Formulation	Aim of Study	Conclusions	Ref.
Fisetin	PVA and PLGA nanoparticles	Optimization of the process and characterization of nanoparticles. Dissolvability and gut permeability test.	A 3.06-fold increase in the dissolution test and a 4.9-fold increase in the permeability test. Developed system improves biopharmaceutical properties.	[78]
Fisetin	Lipid polymer hybrid nanoparticles (LPHNP)	The activity test of fisetin against severe acute pancreatitis (SAP).	Oral LPHNPs loaded with FST protect rats from SAP and multi-organ injury, outperforming fisetin alone, blank LPHNPs, and the untreated group.	[79]
Myricetin	Chitosan nanoparticles	Activity against type 2 diabetes mellitus.	Better glycemic control in an in vivo study. Controlled increase in weight as compared to Metformin. No toxicity or changes in the major organs section in contrast to the normal control	[80]
Quercetin	Zein nanospheres (NS) and zein nanocapsules containing wheat germ oil (NC)	Enhance the bioavailability and efficacy of quercetin.	Similar loading efficiencies and release profiles in simulated fluids. Nanoparticles improved the oral absorption of quercetin in Wistar rats.	[81]
Rutin	Bilosomes	Enhance the renal protective effect of rutin for oral application.	Prolonged release of rutin from bilosomes, relative to free drug. Alleviation of kidney dysfunction, oxidative stress, and inflammation.	[82]
Querceti, fisetin, and rutin	Free form	Determine the photoprotective effects of oral administration of quercetin, fisetin, and rutin, and their accumulation in skin, assessed through mass spectrometry imaging.	Quercetin and fisetin reduced the time to tumor onset, with no observed effect for rutin. Oral administration of quercetin and fisetin to hairless mice increased UVR-induced tumor development.	[83]
Rutin	Rutin hydrate	Iron overload in a genetic mouse model is associated with Type 3 hereditary haemochromatosis patients.	Significant reduction in hepatic ferritin protein expression and serum transferrin saturation. Trends towards decreased iron levels in the liver and serum, and increased serum unsaturated iron binding capacity.	[84]

**Table 5:** Clinical trials using flavonols.

Flavonol	Condition	Aim of the Study	Dosing	Group Size	Age	Results	Ref.
Fisetin	Colorectal cancer	Assessing the effectiveness of supplementation on inflammation and matrix metalloproteinase (MMP) enzyme levels	100 mg/day	42	>40	Reduction in inflammatory markers (IL-8 and hs-CRP); reduction in MMP-7 enzyme (extracellular matrix degradation)	[139]
	Acute ischemic stroke	Lengthening the therapeutic window in the treatment of AIS with rt-PA	100 mg/day	192	>50	Lowering biomarkers of brain damage and inflammation	[140]
	Sepsis	Evaluation of the efficacy of fisetin in preventing clinical deterioration	20 mg/kg	220	>65	-	[141]
Kaempferol	-	Effect of the compound on cardiopulmonary reactions and physical performance	10 mg	17	22	Lower volume of oxygen required (VO2); decreased respiratory rate	[142]
	Rheumatoid arthritis	Explaining the molecular mechanism of RA	5.4 g/day	99	24–75	Improvement in morning stiffness, joint pain and (VAS); reduction in inflammatory markers	[143]
Quercetin	Myocardial infarction	Limiting infarct size in patients with ST-segment elevation myocardial infarction (STEMI)	(500 mg of quercetin and 50 mL 0.9% NaCl)-continuous intravenous infusion	143	18–85	Adding quercetin to standard STEMI therapy reduces infarct size and prevents intramuscular hemorrhage	[144]
	Allergic rhinitis associated with pollinosis	Effect of supplement intake on allergic reactions and quality of life	200 mg/day	66	22–28	Statistically significant reduction in allergic symptoms	[145]
Rutin	T2DM	Effect of supplementation on pancreatic β-cell function and gut microbiota composition	500 mg/day	87	21–64	No significant effect of supplementation on postprandial blood markers and microbiome	[146]

## Data Availability

The datasets used and/or analyzed in the current study are available from the corresponding author upon reasonable request.

## Abbreviations

The following abbreviations are used in this manuscript:

TEAC	Trolox Equivalent Antioxidant Activity
DPPH	2,2-diphenyl-1-picrylhydrazyl radical
topoI	topoisomerase I
topoII	topoisomerase II
ROS	reactive oxygen species
BZF	2-benzoyl-2-hydroxy-3(2H)-benzofuranones
LLC	Lewis lung carcinoma
NLC	nanostructured lipid carriers
ICD	irritant contact dermatitis
DoE	Design of Experiment
SAP	severe acute pancreatitis
NS	nanospheres
NC	nanocapsules
OFAT	one-factor over time
LPHNP	lipid polymer hybrid nanoparticles
SAP	L-arginine-induced acute pancreatitis
T2DM	type 2 diabetes mellitus
SEM	Scanning electron microscope
IBD	inflammatory bowel disease
IgA	immunoglobulin A
PI3K	phosphoinositide 3-kinase
AMPK	5’AMP-activated kinase
G6Pase	glucose-6-phosphatase
PEPCK	phosphoenolpyruvate carboxylase
IBS	irritable bowel syndrome
IFALD	Intestinal Failure associated Liver Disease
FXR	farnesyl receptor
FDA	Food and Drug Administration
LPH	lactase-phlorizin hydrolase enzyme
SGLT1	sodium-dependent glucose transporter
HPLC	high-performance liquid chromatography
TC	total cholesterol
CAD	coronary artery disease
FFQ	food frequency questionnaire
KMP	kaempferol aglycone
TAC	total oxidative capacity
BMF	bone marrow failure
FA	Fanconi anemia
QOL	quality of life
AI	artificial intelligence
TPO	thyroid peroxidase
